# Development of social anxiety cognition scale for college students: Basing on Hofmann’s model of social anxiety disorder

**DOI:** 10.3389/fpsyg.2023.1080099

**Published:** 2023-01-19

**Authors:** Yuxin Zha, Qin Tang, Xiaoru Jin, Xinfei Cai, Wen Gong, Yongcong Shao, Xiechuan Weng

**Affiliations:** ^1^School of Psychology, Beijing Sport University, Beijing, China; ^2^Department of Neuroscience, Beijing Institute of Basic Medical Sciences, Beijing, China

**Keywords:** Hofmann’s model of social anxiety disorder, college students, social anxiety, scale development, emotional control, self-perception, social skills, cost estimation

## Abstract

**Introduction:**

To develop the Chinese version of the Social Anxiety Cognition Scale for College Students (SACS-CS) based on Hofmann’s model of social anxiety disorder and examine its reliability and validity.

**Methods:**

Based on literature analysis and structured interviews, a theoretical model was constructed and behavioral examples were collected. According to the results of participants’ and experts’ evaluations, the initial SACS-CS was developed. The study data were collected from a total of 500 valid participants, randomly divided into two samples. Sample 1 (*n* = 200) and sample 2 (*n* = 300) were considered for exploratory factor analysis and confirmatory factor analysis (CFA), respectively. Internal reliability and validity were examined using all 500 participants, and temporal reliability was established using sample 3 (*n* = 70), who completed the scale again after 4 weeks.

**Results:**

The SACS-CS consists of 21 items, grouped under four factors: self-perception, social skills, emotional control, and cost estimation. The four-factor model fits well. The internal consistency coefficient of the scale and the four factors ranged from 0.87 to 0.96, and the test–retest reliability ranged from 0.76 to 0.84. The scores of the scale and the four factors were significantly correlated with the score of the Interaction Anxiousness Scale (*r* = 0.54–0.64).

**Discussion:**

The SACS-CS possesses good reliability and validity and can be applied in the cognitive assessment of college students’ social anxiety. The scale could help people with different social anxiety disorder conditions receive more personalized interventions.

## 1. Introduction

Anxiety is an unpleasant emotional experience or mental state experienced by individuals. Social anxiety is one of its most common forms (Fernández et al., 2018), which refers to the unreasonable and excessive fear of interpersonal interaction and one’s own performance on social occasions. Specifically, it is manifested as intense nervousness, distress, and a behavioral tendency to avoid social interactions (Bowles, 2017; Van Zalk and Tillfors, 2017; Vassilopoulos et al., 2017). Among college students, most of the people with social phobia suffer from subthreshold social anxiety, which means they avoid public speaking, meeting strangers, or eating in public; this is not associated with significant functional interference (Davidson et al., 1994). Social anxiety disorder (SAD) is only diagnosed if the condition is so severe that its subsequent development strictly meets the diagnostic. If people with subthreshold social anxiety do not receive timely treatment, serious complications may develop, such as social isolation, depression, alcohol addiction, or drug abuse.

In general, the age of individuals suffering from social anxiety varies among children, adolescents and early adulthood. Furthermore, the level of social anxiety in women is significantly higher than that in men (James, 2001). According to the epidemiological survey, SAD is the fourth most common psychological disorder (Michaela et al., 2021), with a lifetime prevalence of 12.1% (Ruscio et al., 2008). The 12-month prevalence was 6.8% in the United States and 2.4% globally (Michaela et al., 2021).

A meta-analysis study showed that the level of social anxiety in Chinese college students increased by year from 1998 to 2015 (Shi et al., 2019). Moreover, according to the studies after 2010, the level of social anxiety in Chinese college students rose (Dong and Zeng, 2020). In China, a study in 2013 (*N* = 905) found that 16% of college students suffered serious social anxiety (Li et al., 2013). A study in 2016 (*N* = 312) found that 22.4% of college students suffered moderate or severe social anxiety (Zhao and Dai, 2016). A small sample study in 2018 (*N* = 167) found that 20.96% of participants met the diagnostic criteria for social anxiety (Li, 2018). A study in 2019 (*N* = 1804) found that 45.7% of college students had social anxiety problems of varying degrees (Li et al., 2019). A study in 2020 (*N* = 820) found that 29.65% of college students had high levels of social anxiety (Zhang et al., 2020). In addition, research by Zhao and Dai (2016) also showed that the average level of social anxiety among Chinese college students was significantly higher than the norm. In summary, the level of social anxiety in Chinese college students was significantly positively correlated with progression of years.

More and more Chinese college students are suffering from social anxiety. They avoid social interaction, fear contact with others as well as others’ evaluation, and suffer from adverse physiological phenomena, such as hand shaking, palpitation, tremor, nausea and so on, which has caused great negative impact on their studies and life. Although existing models have summarized the cognitive characteristics of people with social anxiety, we do not yet know the specific status of different individuals in different cognitive aspects, and we lack the scale to measure the specific status of different cognitive aspects. Therefore, in order to provide personalized CBT for students with social anxiety, we must develop SACS-CS.

Psychological researchers have focused on using psychological measures to diagnose the degree of social anxiety and examining ways to alleviate or even cure it. Early measures of social anxiety appeared in the Multifactor Personality Inventory, which has specific items for measuring social anxiety, although them does not measure it exclusively. An exclusive measurement was created in 1969 (Peng et al., 2004). At present, the main scales widely used internationally include the Social Anxiety subscale of the Self-Consciousness Scale (SASS-CS), Interaction Anxiousness Scale (IAS), Social Avoidance and Distress Scale (SADS), Liebowitz Social Anxiety Scale (LSAS), Social Phobia Scale (SPS), and Social Anxiety Inventory (SAI). Among this, the SASS-CS measures the subjective feelings of anxiety as well as associated verbal and behavioral difficulties. The IAS was developed through a clinical empirical method and mainly measures anxiety feelings in interpersonal communication (Leary, 1983). The SADS was developed by Watson and Friend (1969) and starts with the behaviors and experiences of social anxiety disorder, using rational analysis to establish relevant items to clarify what behaviors are representative of social anxiety. The LSAS is used to assess both fear and avoidance and includes four factors namely, operational fear, operational avoidance, social fear, and avoidance (Liebowitz, 1987). The SPS and SIAS were developed based on the description of social phobia in the Diagnostic and Statistical Manual of Mental Disorders, third edition, revised (DSM-III-R), separately measuring anxiety and fear in the context of interacting with people, performing, or being observed. These two scales are commonly used together (Mattick and Clarke, 1998; Gomez and Watson, 2017). However, each of them has 20 items; thus, it is problematic to apply both in clinical practice due to too many items as participants may feel too tired to complete all the items (Rodebaugh et al., 2006). In order to facilitate clinical application, Fergus et al. (2012) developed the short forms of the Social Interaction Anxiety Scale and the Social Phobia Scale in 2012, which only retained 6 items of each of the original scales and had good reliability and validity. The SAI was based on the social anxiety diagnostic items in the Diagnostic and Statistical Manual of Mental Disorders, fourth edition (DSM-IV), the International Classification of Disease, 10th revision (ICD-10), and the Chinese Classification of Mental Disorders Version 3 (CCMD-3), as well as certain items of the domestic and foreign related scales (Qian et al., 2005).

The aforementioned six scales have been developed by foreign scholars for decades, and most of the social anxiety scales developed in China are translations of foreign ones, which have been tested for reliability and validity. Most of these existing instruments are developed as self-assessment methods of individuals’ feelings and behaviors or directly measure anxiety according to clinical diagnostic criteria. There is a lack of behavioral scales based on sample groups of college students with social anxiety and measuring how they perceive in social interactions. This is necessary to be able to provide more targeted cognitive-behavioral therapy (CBT), which is the most common treatment for people with social anxiety disorder. CBT works by changing patients’ views and attitudes toward people or situations to help them overcome their psychological challenges (Jain et al., 2021). Thus, measuring social anxiety from a cognitive perspective can help understanding sufferers’ cognitive characteristics during social interaction and use these to offer personalized CBT.

In the document retrieval and screening with CBT as the key words, we found that in the cognitive behavioral modification of social anxiety, CBT based on Hofmann’s model has been developed. Hofmann’s model discusses cognitive factors that maintain social anxiety disorder. Studies have demonstrated that conventional CBT principles and general interventions fail to achieve their goals (Hofmann and Otto, 2008), while a 2017 study confirmed that CBT based on Hofmann’s model is a more effective treatment to reduce social anxiety (Roushani et al., 2017). In addition, Neufeld’s study also confirmed that CBT based on Hofmann’s model can effectively reduce social anxiety symptoms and associated psychiatric symptoms (Neufeld et al., 2020). In other words, Hofmann’s model can effectively explain the characteristics of social anxiety and has strong applicability. Therefore, we compared Hofmann’s model with other social anxiety models, and invited six psychological experts to make evaluations on all the selected models. Finally, we adopted the social anxiety disorder model proposed by Hofmann as the theoretical basis to develop the social anxiety scale. Under the advice of experts, the four cognitive characteristics of social anxiety in this model were preliminarily adopted as the four dimensions of the scale, and the determination of these four dimensions were verified in the confirmatory factor analysis. Thus, SACS-CS based on Hofmann’s model could more effectively match the assessment of social anxiety with the intervention.

**Figure 1:**
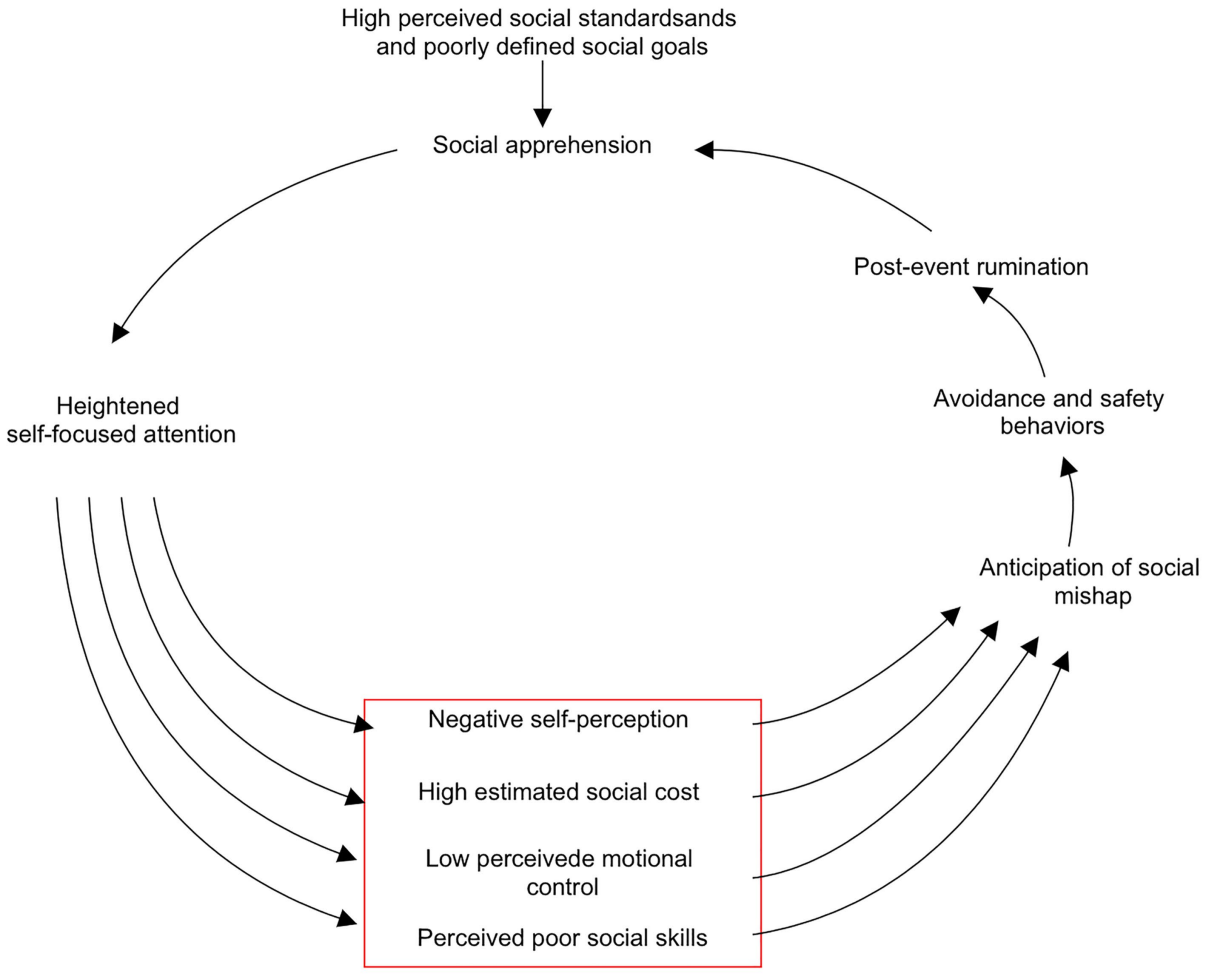
Hofmann’s social anxiety disorder model (The red square highlighted the four cognitive factors) (Hofmann, 2007).

Based on the social anxiety disorder model proposed by Hofmann in 2007, this study developed the Social Anxiety Cognition Scale for College Students (SACS-CS). According to Hofmann’s model (see Figure 1), people with social anxiety disorder (SAD) have higher social standards, they have ambiguous definitions of social goals and are unable to find specific and realistic behavior strategies to achieve them (Hiemisch et al., 2002), which leads to social phobia. Meanwhile, social phobia will also aggravate the cognitive bias of people with SAD (Hofmann, 2007). Social phobia will further increase SAD people’s self-attention, contributing to cognitive characteristics such as negative self-perception, overestimation of social costs, low emotional-control ability, and perception of poor social abilities (Clark and Wells, 1995). These cognitive characteristics will cause them to have catastrophic expectations of social interaction outcomes and adopt avoidance or safety behaviors. Finally, people with SAD will reflect on their behaviors in social conditions, and this process will further intensify their social phobia, creating a vicious cycle (Hofmann, 2007). We selected the cognitive content of this model belonging to the clinical category as the four factors of the SACS-CS scale (as highlighted in the red square of Figure 1): (1) emotional control, referring to difficulty in controlling one’s own emotions or physical reactions, a lack of control that is easily noticed by others; (2) self-perception, referring to thinking that one does not have the characteristics that others expect them to have, underestimating themselves’ abilities, viewing themselves negatively, and expecting others to have a negative viewpoint on them; (3) social skills, referring to being unable to objectively recognize the level of social skills they already have and believing that they lack social skills or are inadequate for social tasks; (4) cost estimation, referring to exaggerating the likeliness of negative events in social situations and overestimating the consequences of such events. We tested the reliability and validity of this scale using a sample of college students.

## 2. Materials and methods

### 2.1. Measurement tools

#### 2.1.1. Social anxiety subscale of the self-consciousness scale

The SASS-CS was developed by Fenigstein, Scheire, and Buss in 1975 and applies to students and adults (Fenigstein et al., 1975). It contains six items that measure not only subjective anxiety, but also behavioral and verbal expression difficulties. Responses were scored on a 5-point scale, ranging from 0 (extreme non-conformity) and 4 (extreme conformity). A score of 24 indicates a high level of anxiety. The Chinese version of this scale has been validated and widely used for the measurement of social anxiety.

#### 2.1.2. Validated measurement tools

The IAS was developed by Leary in 1983 to rate the tendency toward subjective social anxiety, independent of behavior. It consists of 15 self-reported entries, each describing a situation in the first person and asking the respondent to choose the extent to which it corresponds to them. It is a 5-point scale, ranging from 1 (not at all characteristic of me) to 5 (extremely characteristic). Of these, 11 are forward-scoring questions and four are reverse-scoring. Scale scores range from 15 to 75, with social anxiety levels extending from low to high. The Chinese version of IAS showed good reliability and validity in a series of studies.

### 2.2. Measurement process

#### 2.2.1. Item generation

We recruited 30 college students with social anxiety with different majors and grades from different schools and screened them using the SASS-CS. The screening criteria were as follows: (a) self-identified as having social anxiety, (b) scoring 18 or more on the SASS-CS (Fenigstein et al., 1975), (c) identified as socially anxious by a psychological counselor during the interview. We conducted structured interviews with these 30 participants. Based on their reports and the specific performance mentioned in Hofmann’s model for each dimension, we organized the items and developed the initial scale (version 1).

#### 2.2.2. Expert consultation and pilot testing

We then asked 18 participants to evaluate the scale in terms of clarity of presentation and comprehension. Afterward, the initial scale (version 2) was determined. Then, five psychology experts were asked to assess its content validity. Their suggestions included “some items are not measuring cognition” and “there are too many items under one dimension.” In response to these problems, we revised the items and eliminated questions that were not clearly stated. Finally, we ensured that each item measures cognition and the number of items was appropriate under each dimension. This resulted in an initial scale (version 3) with 58 items: 19 for self-perception, 9 for cost estimation, 15 for emotional control, and 15 for social skills.

#### 2.2.3. Sampling and statistical analysis

We combined informed consent (including instructions), personal information (name, gender, school, and contact number), the scale (version 3), a validity criterion (IAS), and two attention check items (e.g., “Please select ‘extremely conforming’“) into one electronic questionnaire with a total of 75 items. The scale factors were randomized with items from different factors appearing randomly instead of in order per factor. Each item described a situation from a first-person perspective and asked the respondent to choose the degree of conformity with their actual situation. The responses were scored on a 5-point scale (1: not at all conforming; 5: extremely conforming).

A total of 830 participants were recruited through the Questionnaire Star platform, each participant received ¥2 in exchange for their participation. After they submitted the survey, we confirmed with each participant whether they answered the items conscientiously. The predetermined screening criteria were as follows: (1) failed the attention check and (2) responded in less than 150 s to all 75 items (Huang et al., 2012). According to the predetermined criteria, 332 participants were excluded. However, after careful confirmation, we included two more participants outside the predetermined criteria who answered the items conscientiously but did not reach the predetermined response time (responded in 142 s and 149 s respectively). The final sample thus comprised 500 participants (154 males and 346 females).

**Figure 2:**
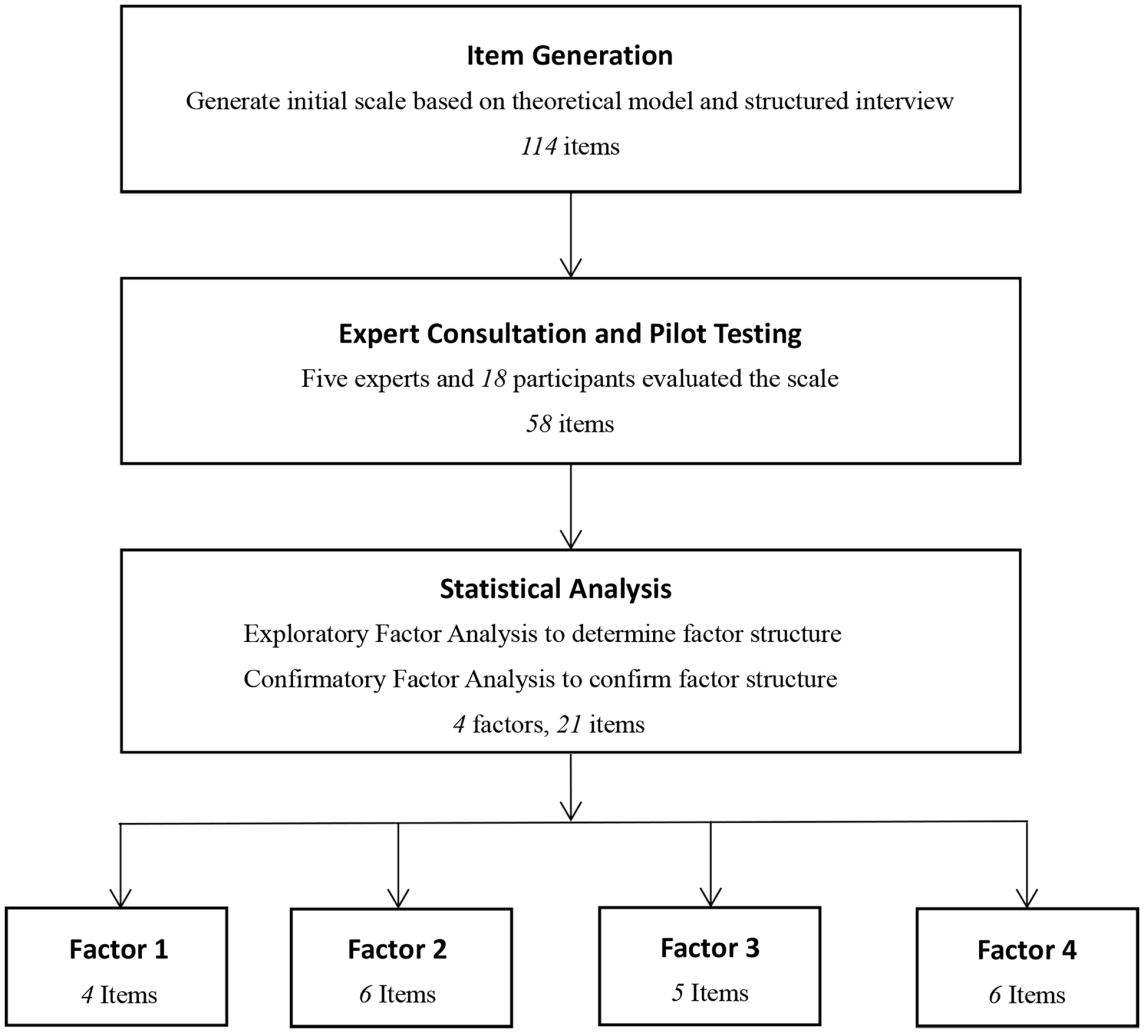
The development of social anxiety cognition scale for college students.

We randomly divided these 500 participants into two samples using random numbers: sample 1 was used (*n* = 200) for exploratory factor analysis (EFA) and sample 2 (*n* = 300) for confirmatory factor analysis (CFA). To test the temporal reliability of the scale, we asked participants who completed the scale on the day of its release to fill it out again 4 weeks later, and 70 (out of 98) participants responded and agreed to refill it and thus formed sample 3 (*n* = 70). To verify its internal consistency, validity, and reliability, we combined the first two samples (*n* = 500). The flowchart of the development of social anxiety cognition scale for college students is shown in Figure 2.

## 3. Results

### 3.1. Items analysis

We performed item analysis by examining the critical ratio and item-total correlation using the data from both samples 1 and 2 (*n* = 500). First, we ranked participants from high to low according to their total score on the scale. The top 27% participants were classified as the high group (*n* = 135), and the bottom 27% participants were classified as the low group (*n* = 135). Then, we used an independent *t*-test to examine the score differences of the two groups for each item. The results showed that responses to the 58 items of the initial scale were significantly different between the two groups (*t* = 10.471–31.731, *p* < 0.001). Furthermore, we checked the item-total correlation using Pearson’s correlation coefficient: all items’ correlation with the total score was significant (*r* = 0.477–0.827, *Ps* < 0.001). The results of the analysis for our final scale comprising 21 items are shown in Table 1.

**Table 1:** The items analysis results for the final SACS-CS.

Items		*t*	*r*
2	When socializing, I always assume that others will comment negatively on my image.	24.91	0.77
3	I always think that my image is annoying or uncomfortable for others.	17.90	0.71
7	I always think that others will not recognize my ability.	23.21	0.79
11	When socializing, I always believe that others will dislike my disposition.	27.40	0.80
23	I always believe that I am going to speak or behave improperly.	24.62	0.77
36	When socializing, I always think I will do something that cannot be explained afterward.	17.73	0.65
25	Whenever something bad happens in social situations, I think it will have unacceptable consequences.	14.31	0.58
26	I always believe that, if I do or say something wrong, it will lead to serious consequences.	25.19	0.75
27	I always think that, once I fail in a social interaction, I will leave a bad impression to others.	28.12	0.77
28	If I receive an unfavorable evaluation, I think that everyone present will always remember it.	22.27	0.71
31	When socializing, I do not think I can control my nervousness.	26.63	0.80
32	When socializing, I think it’s hard to relieve my tension no matter how I try.	19.76	0.71
33	When socializing, I always think I cannot control my physiological responses (such as blushing, shaking).	19.56	0.69
35	When socializing, I think I may have uncontrollable behaviors due to nervousness or fear (such as picking fingers).	17.74	0.67
43	When socializing, I think people can easily tell that I ‘m uncontrollably nervous by my facial expressions.	18.79	0.68
37	I always think that I have no control over the direction of social interaction (such as topics, atmosphere).	21.55	0.75
45	I always think I’m unable to communicate with others.	23.41	0.77
47	I think my social skills are very poor.	26.59	0.77
48	I think I often fall into awkward situations when communicating with others.	21.37	0.73
51	I do not think I can handle social situations alone.	22.82	0.77
53	I think there is no situation where I can adequately use my social skills.	15.99	0.64

### 3.2. Exploratory factor analysis

The data from sample 1 (*n* = 200) were used to complete EFA on the initial 58 items (model A). We first used the principal component analysis method to extract four factors with eigenvalues greater than 1 and used the maximum variance method to perform the orthogonal rotation. Then, according to Hofmann’s theoretical model and the loading value of each item, we deleted the items that did not conform to the following criteria: (a) the biggest loading value of the item is greater than 0.5, (b) the loading value of the item is less than 0.5 on two or more factors, and (c) the factor of the item conform to the theoretical model. We deleted the items one at a time, 31 items in total. Finally, we obtained model B consisting of 27 items on four factors. The EFA of model B showed that the Kaiser-Meyer-Olkin (KMO) was 0.962, Bartlett ‘s sphericity test was significant (*χ*^2^_(351)_ = 4183.46，*p* < 0.001), items in model B accounted for 68.691% of the total variance, and the loading value of each item was between 0.54 and 0.78. We named the factors according to our theoretical model: factor 1, self-perception, consists of eight items and has an eigenvalue of 14.803; factor 2, social skills, includes seven items and has an eigenvalue of 1.434; factor 3, emotional control, consists of six items with an eigenvalue of 1.299; factor 4, cost estimation, consists of six items, with an eigenvalue of 1.012 (Table 2).

**Table 2:** Exploratory factor analysis of SACS-CS.

Items	Factor loading			
	1	2	3	4
Factor 1: self-perception				
7. I always think that others will not recognize my ability.	**0.685**	0.367	0.215	0.361
3. I always think that my image is annoying or uncomfortable for others.	**0.680**	0.184	0.424	0.184
2. When socializing, I always assume that others will comment negatively on my image.	**0.667**	0.227	0.242	0.405
11. When socializing, I always believe that others will dislike my disposition.	**0.656**	0.283	0.352	0.32
1. When socializing with others, I always think my image is not good enough.	**0.646**	0.208	0.391	0.277
6. When socializing, I always think others will question my ability.	**0.632**	0.375	0.109	0.403
12. I think my disposition is very bad in other’s view.	**0.604**	0.354	0.348	0.172
14. When socializing, I always think that other’s will give me negative comments.	**0.564**	0.314	0.047	0.440
Factor 2: social skills				
47. I think my social skills are very poor.	0.332	**0.777**	0.217	0.139
48. I think I often fall into awkward situations when communicating with others.	0.148	**0.762**	0.215	0.313
45. I always think I’m unable to communicate with others.	0.434	**0.688**	0.228	0.246
37. I always think that I have no control over the direction of social interaction (such as topics, atmosphere).	0.344	**0.687**	0.244	0.256
53. I think there is no situation where I can adequately use my social skills.	0.139	**0.641**	0.317	0.257
46. I think my social skills are not enough for daily communication.	0.476	**0.563**	0.298	0.227
51. I do not think I can handle social situations alone.	0.302	**0.543**	0.445	0.298
Factor 3: emotional control				
34. When socializing, I think I will have uncontrollable hand movements (shaking hands or fiddle with small objects).	0.148	0.156	**0.795**	0.228
35. When socializing, I think I may have uncontrollable behaviors due to nervousness or fear (such as picking fingers).	0.214	0.218	**0.738**	0.235
33. When socializing, I always think I cannot control my physiological responses (such as blushing, shaking).	0.252	0.339	**0.715**	0.170
43. When socializing, I think people can easily tell that I ‘m uncontrollably nervous by my facial expressions.	0.214	0.248	**0.683**	0.296
31. When socializing, I do not think I can control my nervousness.	0.431	0.327	**0.612**	0.284
32. When socializing, I think it’s hard to relieve my tension no matter how I try.	0.412	0.246	**0.557**	0.247
Factor 4: cost estimation				
26. I always believe that, if I do or say something wrong, it will lead to serious consequences.	0.283	0.277	0.131	**0.747**
28. If I receive unfavorable evaluation, I think that everyone present will always remember it.	0.236	0.186	0.278	**0.732**
27. I always think that, once I fail in a social interaction, I will leave a bad impression to others.	0.287	0.316	0.250	**0.677**
23. I always believe that I am going to speak or behave improperly.	0.281	0.187	0.383	**0.621**
36. When socializing, I always think I will do something that cannot be explained afterward.	0.185	0.228	0.344	**0.600**
25. Whenever something bad happens in social situations, I think it will have unacceptable consequences.	0.250	0.152	0.199	**0.587**

Factor loadings above 0.5 are in bold.

### 3.3. Confirmatory factor analysis

**Figure 3:**
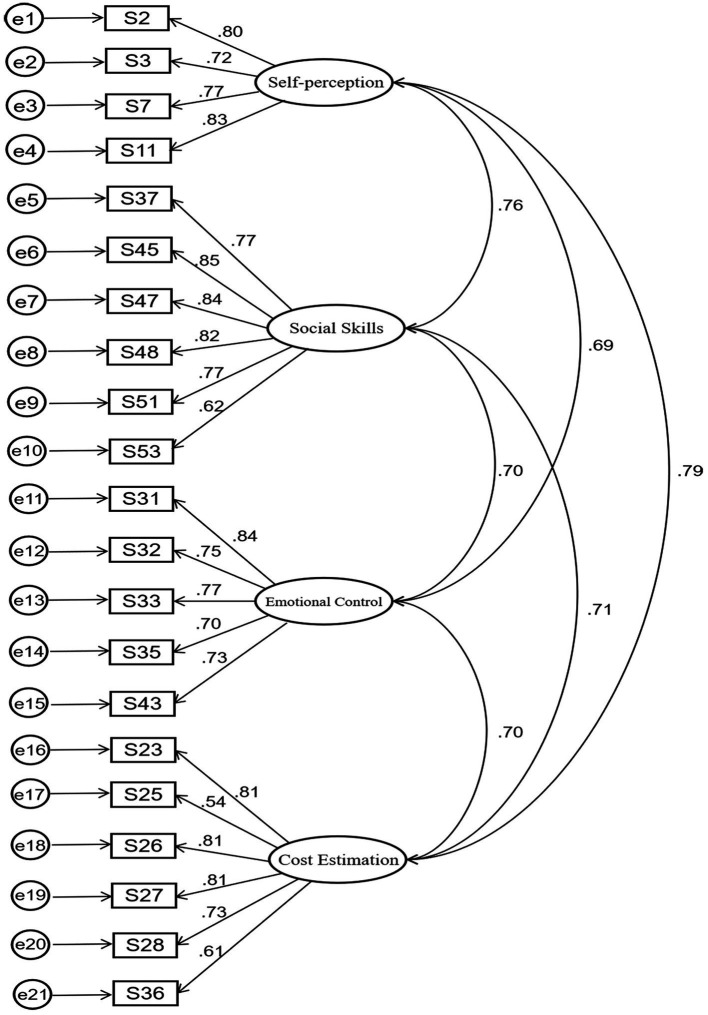
Confirmatory factor analysis for Model C.

After the EFA, a 27-item SACS-CS was finally formed. To investigate the degree of fitness between the conceived model and the actual one, we conducted CFA on model B. Sample 2 (*n* = 300) was used to examine the construct validity in the four dimensions formed by the EFA, with each dimension as a latent variable. We examined the pairwise correlations between the four latent variables, and the residuals of 27 measured variables were set as independent. The fitting index of model B is shown in Table 3. It can be seen that the fitting of the data and model B is essentially good. Since the root-mean-square error of approximation (RMSEA) value was slightly high, further reduction was made according to the item factor loadings. Items 1, 6, 12, 14, 34, and 46 with double loads were deleted, and model C of the remaining 21 items was subjected to CFA again (see Figure 3).

**Table 3:** Fitting index for confirmatory factor analysis of Model B & C.

Model	χ^2^/df	RMSEA	SRMR	GFI	AGFI	NFI	CFI	TLI
Model B	2.594	0.073	0.045	0.83	0.79	0.86	0.91	0.90
Model C	1.855	0.053	0.037	0.90	0.88	0.92	0.96	0.96

The revised model C specifies four factors (factor 1 consists of four items, factor 2 consists of six items, factor 3 consists of five items, factor 4 consists of six items). The factor loading showed the following: the *χ*^2^ (df = 203, *p* < 0.01) was 339.440, comparative fit index (CFI) was 0.96, RMSEA was 0.053 (90%CI 0.045–0.062), and standardized residual mean root (SRMR) was 0.037. In addition, each of the 21 items had moderate and high factor loadings (0.54–0.85), indicating that the data were highly correlated with model C (Tables 3, 4).

**Table 4:** Factor loading of social anxiety cognition scale for college students.

Item	Factor 1	Item	Factor 2	Item	Factor 3	Item	Factor 4
S2	0.80	S37	0.77	S31	0.84	S23	0.81
S3	0.72	S45	0.85	S32	0.75	S25	0.54
S7	0.77	S47	0.84	S33	0.77	S26	0.81
S11	0.83	S48	0.82	S35	0.70	S27	0.81
		S51	0.77	S43	0.73	S28	0.73
		S53	0.62			S36	0.61

### 3.4. Reliability

We calculated the Cronbach’s alpha of each factor and of the total scale using the data from both samples 1 and 2 (*n* = 500) to examine the internal consistency of the scale. The results demonstrated the reliability of the scale: *α*_factor 1_ = 0.88, *α*_factor 2_ = 0.91, *α*_factor 3_ = 0.88, *α*_factor 4_ = 0.87. The Cronbach’s alpha of the final SACS-CS was 0.96.

We selected participants who completed the scale on the day of its release to fill it out again 4 weeks later (sample 3, *n* = 70). We used sample 3 to examine the test–retest reliability of the SACS-CS. We calculated the correlation coefficient between two measurements for each factor and the total score. The results showed that the scale had good temporal stability (*r* > 0.75): *r*_factor 1_ = 0.79, *r*_factor 2_ = 0.81, *r*_factor 3_ = 0.77, *r*_factor 4_ = 0.76. The test–retest reliability of the total scale was 0.84. Therefore, the final scale has good stability and consistency across time.

### 3.5. Validity

To evaluate the degree of consistency between each dimension and the total scale, the correlation matrix was calculated. It can be seen from the results that there is a significant correlation between the score of the total scale and of each dimension. The correlation coefficient *r* was between 0.87 and 0.90. There was also a significant correlation between the scores of all dimensions; the correlation coefficient *r* was between 0.69 and 0.79. The correlation between the total scale score and the score of each dimension of the SACS-CS was higher than the correlation between the scores of all dimensions, indicating that the latter has a strong attribution to the total scale but are also independent from each other.

To assess the criterion validity of the scale, we examined the correlation between SACS-CS and IAS, which is commonly used to measure the degree of social anxiety. We expected that all four factors and the total scores of the scale would be significantly correlated with the IAS. The Pearson correlation analysis demonstrated that the scores of the SACS-CS and IAS had a significant positive correlation. The scores of self-perception (factor 1), social skills (factor 2), emotional control (factor 3), and cost estimation (factor 4) were also significantly positively correlated with IAS scores. The correlation coefficient *r* was between 0.54 and 0.64 (*p* < 0.001). Since the SACS-CS and IAS both measure the degree of individual social anxiety, the significant moderate strength correlation between the two scales indicated that the SACS-CS has good criterion validity (see Table 5).

**Table 5:** Correlation matrix between SACS-CS and IAS.

Variable	*n*	*M*	SD	SP	SS	EC	CE	Total	IAS
SP	500	10.03	3.86	1					
SS	500	16.24	5.97	0.76^**^	1				
EC	500	13.76	4.89	0.69^**^	0.70^**^	1			
CE	500	17.70	5.70	0.79^**^	0.71^**^	0.70^**^	1		
total	500	57.73	18.19	0.89^**^	0.90^**^	0.87^**^	0.90^**^	1	
IAS	500	44.10	8.53	0.54^**^	0.57^**^	0.59^**^	0.58^**^	0.64^**^	1

**p < 0.001. SACS-CS, Social Anxiety Cognition Scale for College Students; SP, self-perception; SS, social skills; EC, emotional control; CE, cost estimation.

## 4. Discussion

Compared to Chinese studies, foreign studies on social anxiety measurement started earlier and produced more results. For example, the first scale assessing social anxiety, the Social Avoidance and Distress Scale (SAD), was developed by Watson and Friend, (1969), while Chinese scales primarily formed through translation and indigenization of foreign scales, especially from the early years. While a few researchers developed some original social anxiety scales (e.g., Zhao, 2004), most could not been popularized and used in China.

Despite the differences between starting time and research quantities, the scope and content of the two studies are the same. The studies primarily focused on three aspects: subjective perception, cognition, and behavior. For example, the Interaction Anxiousness Scale (IAS) focused on subjective experiences, while the Social Phobia Scale (SPS) focused on subjective experiences and behavioral performances. Meanwhile, we found few scales measuring the cognitive aspects related to social anxiety, such as the Negative Cognitive Processing Bias Questionnaire (NCPBQ; Yan et al., 2017). NCPBQ is based on Beck’s negative cognitive processing bias theory, contains four dimensions: negative attention bias, negative memory bias, negative interpretation bias, and negative rumination bias. However, this scale was not developed under the premise of social anxiety and does not contain contents of maladaptive cognition, which is a key factor causing social anxiety.

In recent years, researchers have paid more attention to social anxiety, and the number of published studies has been rising (Li et al., 2022). However, the targeted populations have been shifting—from the initial adult group to children and teenagers, especially college students. Some researchers in China developed social anxiety scales for college students (e.g., Qian et al., 2005). Simultaneously, we found that further research is needed on prevention and treatment of social anxiety. Therefore, in addition to exploring the structure of college students’ social anxiety cognition, this study assisted the prevention and treatment of social anxiety. Compared to other scales’ objectives that focused more on identifying socially anxious people, this scale aims more at helping socially anxious people understand their maladaptive cognition to facilitate the treatment, especially for Internet-based Cognitive Behavioral Therapy (ICBT).

In our study, we adopted Hofmann’s model of social anxiety disorder (2007), aiming at the cognitive aspect of social anxiety. Combining the theory with the characteristics of Chinese college students, we determined the four-factor model of cognitive social anxiety: self-perception, social skills, emotional control, and cost estimation and developed SACS-CS. The results have shown that the cognition of social anxiety among Chinese college students fits well with the theoretical model, ensuring that the scale conforms with the theory as well as Chinese culture.

This scale consists of 21 items. Its Cronbach’s alpha is greater than 0.9, suggesting that it has a good internal consistency. As seen in Table 2, the eigenvalue for each factor is greater than 1 and the loading value of each item is between 0.54 and 0.78, accounting for 68.691% of the total variance. The results in Table 5 suggest that the correlations between all factors are between 0.69 and 0.79, and the factor-total correlation is between 0.87 and 0.90. The correlations between all factors are less than the factor-total correlation, which suggests that each dimension is relatively independent. Thus, the scale has good construct validity. The CFA results show that CFI > 0.95, RMSEA <0.06, SRMR <0.05, suggesting that the data were highly correlated with the four-factor model of the SACS-CS. Therefore, the SACS-CS has good reliability and validity and is suitable for identifying college students ‘cognitive perspective of social anxiety.

### 4.1. Theoretical implications

Cognitive models of social anxiety emphasizes the role of cognition in the formation of social anxiety, implying that maladaptive cognitions during social situations are the main factors leading to social anxiety (Hyett and Mcevoy, 2017). However, the most existing social anxiety scales are compiled from the perspectives of behavioral patterns and subjective feeling, while fewer scales were focusing on the cognitive aspects of social anxiety. Nevertheless, our scale measures anxiety from a cognitive perspective, providing a novel and useful tool for measuring social anxiety. The study chooses IAS, a commonly used scale measuring social anxiety from the perspective of subjective feeling, as a criterion. The Pearson correlation analysis has shown that SACS-CS and IAS had a significant positive correlation (*r* = 0.64). This demonstrated that the cognitive factors SACS-CS measures are highly correlated with subjective social anxiety, indicating a strong link between inaccurate cognition and social anxiety.

Furthermore, we found that Hofmann’s model of SAD (2007) had great reference value. Through EFA and CFA, the study has verified the four-factor model of social anxiety’s cognitive triggers: self-perception, social skills, emotional control, and cost estimation, which is consistent with the four cognitive factors stated in Hofmann’s model. The results from EFA demonstrated that self-perception dimension had an eigenvalue of 14.803, which was much higher than other dimensions (1.012–1.434), indicating that self-perception may be a key factor affecting social anxiety. The results from CFA have shown that the model’s overall fit was good, indicating that this four-factor model is suitable for the cognitive assessment of social anxiety among college students.

### 4.2. Practical implications

The aggravation of social anxiety can lead to severe SAD (Iverach and Rapee, 2014). College students are more likely to suffer from SAD due to high social demands and academic pressure, which may impair their learning, social function, physical health, and the quality of life (Wang et al., 2016; Creswell et al., 2020). A longitudinal study of participants with social anxiety, with an average age of 19, showed that only 37% of them recovered in the next 12 years (Eleanor and David, 2018). Therefore, prevention and treatment of social anxiety should be taken seriously.

Current treatment alternatives include CBT (Powers et al., 2010), Attention Bias Modification (ABM; Dandeneau and Baldwin, 2005), Interpretation Bias Modification (IBM; Brettschneider et al., 2015), and Systemic Desensitization Therapy (SDT; Goudemand et al., 1980). Among these treatments, CBT is widely used and has achieved good results in clinical practice. A study comparing the efficacy of phenelzine and CBT to that of placebo has shown that, although the two treatment groups achieved the same results, after 1 year, the drug treatment group had a recurrence rate of 50%, whereas the CBT one only had a recurrence rate of 17% (Heimberg et al., 1998). This has demonstrated the effectiveness of CBT; however, applying it to different types of social anxiety with different levels of severity requires practitioners to spend a considerable amount of time to design a customized plan specific to the patient’s needs. There are also some difficulties in applying CBT on a large scale, such as high expenses and the insufficiency of therapists (van Straten and Cuijpers, 2009). This study aimed to enhance the understanding of college students’ social anxiety cognitions, reduce the cost of CBT, and facilitate its implementation. Compared to previous scales, the scale reflects a multi-dimensional construction of college students’ social anxiety cognition basing on Hofmann’s model of SAD. The results on its different dimensions can be analyzed and interpreted to help understand the specific cognitive abnormality of socially anxious patients. This means that more effective CBT programs can be developed with the help of our scale.

In addition, some studies have explored the possibility of providing self-help psychotherapy through smart phones, which is defined as Internet-based Cognitive Behavioral Therapy (ICBT; Norris et al., 2013; Dagöö et al., 2014). ICBT, derived from the theory and technique of CBT, is a self-help online reading and experiencing therapy basing on a safe internet platform with the help from professional clinical therapists (Furmark et al., 2009). In the past decade, ICBT has been developed continuously and has been shown to effectively improve the social anxiety symptoms of socially anxious patients. Previous studies in China also show that localized ICBT can effectively improve social anxiety symptoms of Chinese participants (Kishimoto et al., 2016; Chen et al., 2020). At the same time, ICBT courses based on this have also been developed whereby patients can learn to independently conduct their own CBT. In this way, patients who are aware of their cognitive abnormality through SACS-CS can choose to focus on more specific courses addressed to their cognitive problems when conducting ICBT.

### 4.3. Limitations and future directions

Although the SACS-CS has been shown to have good reliability and validity, some of its limitations should also be considered. First, the proportions of male and female participants were unbalanced, which affected the representativeness of college students in this research to a certain extent. Future studies may choose a better way to collect data, such as stratified sampling, to balance the gender proportions and other demographic factors of the participants. Second, this study lacked some potentially useful demographic information, such as family backgrounds. Future studies may collect more information in the survey to discuss more detailed individual differences. Third, this study only considered four cognitive factors from Hofmann’s model. Although this included the most typical factors, as the diversity of people’s cognition, more cognitive factors may need to be considered. Future studies should integrate more cognitive models and practical experiences, explore other cognitive factors that may contribute to social anxiety, thereby improving the current scale. Furthermore, whether the foreign theoretical model of social anxiety is suitable for Chinese people’s assessment should also be considered in future studies.

If this last fact can be established, the next steps may be increasing the sample size and balancing the gender proportions of the participants as well as expanding the scope of application of the scale to further examine its reliability and validity. In addition, the content of each dimension in this scale is different. In the future, more in-depth research on a single dimension of the SACS-CS can be conducted and a more specialized scale for each dimension can be created. At the same time, the measurement of the SACS-CS has implications for the diagnosis of social anxiety, which can enable therapists to better understand the cognitive status of their social anxiety patients and customize CBT accordingly. However, the impact of social anxiety cognition on individual emotions and behaviors still need more empirical research. Future research should consider that the scale was developed and validated in the context of China and the English version needs to be further revised for a Western sample. In conclusion, the SACS-CS performs well on a psychometric level. Compared with previous scales, the SACS-CS has more detailed and comprehensive contents, which compensates for its limitations.

## 5. Conclusion

Basing on structured interview and Hofmann’s Model of Social Anxiety Disorder, the study developed SACS-CS and conducted EFA and CFA to verify its reliability and validity. The results have shown that SACS-CS possesses good reliability and validity and can be applied in the cognitive assessment of college students’ social anxiety. The study has proven the four-factor model of cognitive social anxiety: emotional control, self-perception, social skills, and cost estimation. The scale can enhance the understanding of college students’ social anxiety cognition, facilitate the implementations of cognitive behavioral therapy, and help college students better conduct internet-based behavioral therapy.

## Data availability statement

The raw data supporting the conclusions of this article will be made available by the authors, without undue reservation.

## Ethics statement

Ethical review and approval was not required for the study on human participants in accordance with the local legislation and institutional requirements. The patients/participants provided their written informed consent to participate in this study.

## Author contributions

YZ designed the project, lead the data collection, conducted the analyzes, and wrote the results section. QT lead the data collection and wrote the introduction section. XJ lead the data collection and wrote the results section. XC lead the data collection and wrote the Method section. WG lead the data collection and wrote the discussion section. YS and XW reviewed the literature and revised the manuscript. All the authors contributed substantially to the manuscript and approved the content of the manuscript.

## Funding

This work was financially supported by the National Natural Science Foundation of China (Grant Number 82073833).

## Conflict of interest

The authors declare that the research was conducted in the absence of any commercial or financial relationships that could be construed as a potential conflict of interest.

## Publisher’s note

All claims expressed in this article are solely those of the authors and do not necessarily represent those of their affiliated organizations, or those of the publisher, the editors and the reviewers. Any product that may be evaluated in this article, or claim that may be made by its manufacturer, is not guaranteed or endorsed by the publisher.
