# Supplementary material for: Development of social anxiety cognition scale for college students: Basing on Hofmann’s model of social anxiety disorder
Source: Front Psychol. 2023 Jan 19;14:1080099. doi: 10.3389/fpsyg.2023.1080099 (PMC9892844; doi:10.3389/fpsyg.2023.1080099)
Supplement: Supplementary file 1 [file Table_1.docx]

## **Supplementary Material 1 |** Social Anxiety **C**ognition Scale (SACS-CS)

| Factors | No. | Items |
| --- | --- | --- |
| Self-  perception | 2 | When socializing, I always assume that others will comment negatively on my image. |
|  | 3 | I always think that my image is annoying or uncomfortable for others. |
|  | 7 | I always think that others will not recognize my ability. |
|  | 11 | When socializing, I always believe that others will dislike my disposition. |
| Cost Estimation | 23 | I always believe that I am going to speak or behave improperly. |
|  | 25 | Whenever something bad happens in social situations, I think it will have unacceptable consequences. |
|  | 26 | I always believe that, if I do or say something wrong, it will lead to serious consequences. |
|  | 27 | I always think that, once I fail in a social interaction, I will leave a bad impression to others. |
|  | 28 | If I receive an unfavorable evaluation, I think that everyone present will always remember it. |
|  | 36 | When socializing, I always think I will do something that cannot be explained afterward. |
| Emotional Control | 31 | When socializing, I don't think I can control my nervousness. |
|  | 32 | When socializing, I think it's hard to relieve my tension no matter how I try. |
|  | 33 | When socializing, I always think I can't control my physiological responses (such as blushing, shaking). |
|  | 35 | When socializing, I think I may have uncontrollable behaviors due to nervousness or fear (such as picking fingers). |
|  | 43 | When socializing, I think people can easily tell that I 'm uncontrollably nervous by my facial expressions. |
| Social Skills | 37 | I always think that I have no control over the direction of social interaction (such as topics, atmosphere). |
|  | 45 | I always think I'm unable to communicate with others. |
|  | 47 | I think my social skills are very poor. |
|  | 48 | I think I often fall into awkward situations when communicating with others. |
|  | 51 | I don't think I can handle social situations alone. |
|  | 53 | I think there is no situation where I can adequately use my social skills. |
