# Supplementary material for: Development of social anxiety cognition scale for college students: Basing on Hofmann’s model of social anxiety disorder
Source: Front Psychol. 2023 Jan 19;14:1080099. doi: 10.3389/fpsyg.2023.1080099 (PMC9892844; doi:10.3389/fpsyg.2023.1080099)
Supplement: Supplementary file 2 [file Table_2.docx]

# **Supplementary Material 2 | Questions in the Structured Interviews**

| No. | Items |
| --- | --- |
| 1 | When you socialize with others, do you pay special attention to their tone, expressions and actions? |
| 2 | What kind of tone, expressions and actions are you sensitive about? |
| 3 | Are there any other details in social situations you pay attention to? |
| 4 | What situation makes you feel your anxiety level rises rapidly? Can you describe the situation? |
| 5 | When you focus on certain social details, do you focus on them continuously? Can you give some examples? |
| 6 | When facing situations that cause your anxiety, do you immediately avoid them? (examples) |
| 7 | What details do you avoid when you notice them? Why? |
| 8 | Can you feel your emotions when socializing with others? What kind of emotions? |
| 9 | When socializing, can you feel changes in your body? What are the changes? |
| 10 | What thoughts do you have when socializing with others? |
| 11 | When someone changes their facial expression or posture while talking to you, do you have any negative thoughts? |
| 12 | What do you think when others say something that seems neutral? (examples) |
| 13 | When socializing, do you interpret negatively ambiguous social situations? (examples) |
| 14 | Before socializing, do you have negative expectations about your performance? What are they? What do you expect others to comment about you? What differences do you see between how others see you and how others expect you to be? Do you have negative emotions, such as anxiety, because you are afraid you cannot meet others' expectations? What kind of emotions? |
| 15 | Before socializing, do you have negative expectations about the future social encounters or their outcome? What are they? (examples) |
| 16 | Are you afraid of others' comments? |
| 17 | After a social failure, do you attribute it to yourself or your behavior? How do you usually explain your failures to yourself? |
| 18 | When facing negative social outcomes, do you always blame yourself and feel ashamed? If so, can you give an example? |
| 19 | After socializing, what kinds of facial expressions, attitudes, behaviors, comments, words etc. of others do you remember the most? |
| 20 | After socializing, what kinds of attitudes, behaviors, emotions, thoughts, feelings etc. of yourself do you remember the most? |
| 21 | After socializing, what kind of situation do you remember the most? |
| 22 | After socializing, do you keep recalling the social experiences? What kinds of experiences do you recall, and for how long? |
| 23 | When recalling a social experience, do you think actively? What do you think? (for example, do you think about how to avoid your behavior and feelings? Or do you reflect on the mistakes you made?) Do you feel better about the event after thinking about it? |
| 24 | In addition to active recalling, have you ever experienced passive recalling? (for example, you do not want to think about certain social events anymore, but memories and thoughts keep coming back to you). If so, what kind of experiences? |
| 25 | Is your recalling more active or passive? |
